# Improved and standardized method for assessing years lived with disability after burns and its application to estimate the non-fatal burden of disease of burn injuries in Australia, New Zealand and the Netherlands

**DOI:** 10.1186/s12889-020-8233-8

**Published:** 2020-01-29

**Authors:** Inge Spronk, Dale W. Edgar, Margriet E. van Baar, Fiona M. Wood, Nancy E. E. Van Loey, Esther Middelkoop, Babette Renneberg, Caisa Öster, Lotti Orwelius, Asgjerd L. Moi, Marianne Nieuwenhuis, Cornelis H. van der Vlies, Suzanne Polinder, Juanita A. Haagsma

**Affiliations:** 1 Erasmus MC, Department of Public Health, University Medical Center Rotterdam, P.O. Box 2040, 3000 CA Rotterdam, The Netherlands; 2 Association of Dutch Burn Centres, Maasstad Hospital, Rotterdam, the Netherlands; 3 Amsterdam UMC, Department of Plastic, Reconstructive and Hand Surgery, Amsterdam Movement Sciences, Vrije Universiteit Amsterdam, Amsterdam, Netherlands; 4 State Adult Burn Unit, Fiona Stanley Hospital, Murdoch, Western Australia Australia; 5 Burn Injury Research Node, The University of Notre Dame, Fremantle, Western Australia Australia; 6 Fiona Wood Foundation, Murdoch, Western Australia Australia; 7 Association of Dutch Burn Centres, Department Behavioural Research, Beverwijk, the Netherlands; 8 Department Clinical Psychology, Utrecht University, Utrecht, the Netherlands; 9 Association of Dutch Burn Centres, Red Cross Hospital, Beverwijk, the Netherlands; 10 Department of Clinical Psychology and Psychotherapy, Freie Universität Berlin, Berlin, Germany; 11 Department of Neuroscience, Psychiatry, Uppsala University, Uppsala, Sweden; 12 Department of Anaesthesiology and Intensive Care, and Department of Clinical and Experimental Medicine, Linköping University, Linköping, Sweden; 13 Department of Health and Caring Sciences, Faculty of Health and Social Sciences, Western Norway University of Applied Sciences, Bergen, Norway; 14 Department of Plastic, Hand and Reconstructive Surgery, National Burn Centre, Haukeland University Hospital, Bergen, Norway; 15 Association of Dutch Burn Centres, Martini Hospital, Groningen, the Netherlands; 16 Center for Human Movement Sciences, University of Groningen, University Medical Center Groningen, Groningen, The Netherlands; 17 Burn Centre, Maasstad Hospital, Rotterdam, the Netherlands; 18 Trauma Research Unit Department of Surgery, Erasmus MC, University Medical Center Rotterdam, Rotterdam, The Netherlands

**Keywords:** Burden of disease, Burn injuries, Methodology, Disability weights

## Abstract

**Background:**

Burden of disease estimates are an important resource in public health. Currently, robust estimates are not available for the burn population. Our objectives are to adapt a refined methodology (INTEGRIS method) to burns and to apply this new INTEGRIS-burns method to estimate, and compare, the burden of disease of burn injuries in Australia, New Zealand and the Netherlands.

**Methods:**

Existing European and Western-Australian health-related quality of life (HRQL) datasets were combined to derive disability weights for three homogenous burn injury groups based on percentage total body surface area (%TBSA) burned. Subsequently, incidence data from Australia, New Zealand, and the Netherlands from 2010 to 2017 were used to compute annual non-fatal burden of disease estimates for each of these three countries. Non-fatal burden of disease was measured by years lived with disability (YLD).

**Results:**

The combined dataset included 7159 HRQL (EQ-5D-3 L) outcomes from 3401 patients. Disability weights ranged from 0.046 (subgroup < 5% TBSA burned > 24 months post-burn) to 0.497 (subgroup > 20% TBSA burned 0–1 months post-burn). In 2017 the non-fatal burden of disease of burns for the three countries (YLDs/100,000 inhabitants) was 281 for Australia, 279 for New Zealand and 133 for the Netherlands.

**Conclusions:**

This project established a method for more precise estimates of the YLDs of burns, as it is the only method adapted to the nature of burn injuries and their recovery. Compared to previous used methods, the INTEGRIS-burns method includes improved disability weights based on severity categorization of burn patients; a better substantiated proportion of patients with lifelong disability based; and, the application of burn specific recovery timeframes. Information derived from the adapted method can be used as input for health decision making at both the national and international level. Future studies should investigate whether the application is valid in low- and middle- income countries.

## Background

A well-established concept that assesses the impact of a health problem or disease on a population is burden of disease. Since the Global Financial Crisis, funding for health care is scrutinised carefully and increasingly objective justification is required for spending within contemporary political systems. Priority setting in health care, surveillance, interventions and resource allocation is based increasingly on burden of disease and injury studies. Burden of disease comprises all health consequences of a disease or injury in one metric: the disability adjusted life year (DALY), which allows comparison across diseases and injuries, populations, and over time [1–3]. This metric combines information regarding loss of health due to premature mortality with information on non-fatal disabling effect of diseases and/or injuries in one single figure [4–6]. Premature mortality is expressed as years of life lost (YLLs) and non-fatal health loss as years lived with disability (YLDs), which are adjusted for the severity of the disability [7, 8]. YLDs for a given disease or injury are derived by multiplying the incidence of the disease or injury by a disability weight. A disability weight reflects the magnitude of health loss and has a value between zero and one, with zero for perfect health and one for a health state that is equivalent to death [1, 9]. Combining information regarding the duration of the disability with the disease or injury specific disability weights is necessary to derive adequate YLDs and consequently DALYs [10].

Due to a great variety of outcomes after a single type of injury, which may vary between mild to very serious sequelae [10], there was a mismatch between injury incidence data and disability weights [11]. To overcome this problem, a refined method, the INTEGRIS method, to assess the non-fatal burden of injury was developed by Haagsma et al. [10]. This method improved the linkage between injury incidence data and injury disability weights by taking into account the heterogeneity among nature-of-injury groups. Important adaptations in this novel method are: 1) a more detailed classification of injuries to obtain more homogenous severity categories, 2) an extension of the number of short and long-term disability weights for consequences of injuries, and 3) assessment of the proportion of patients with a permanent disability based on empirical population data instead of expert opinion [10]. The method consists of three steps: 1) data collection on the incidence and age distribution of the studied injury; 2) breaking down incidence data into homogenous injury categories at functional level; 3) combination of the grouped incidence data with disability weights the estimated duration of the disability [10].

Although a vast improvement over previous methods, application of this refined method in the field of burn injury is still difficult as there is no granular classification of severity of burn injuries to obtain homogenous burn injury groups. Burns are a major cause of mortality and morbidity worldwide. As estimated by the World Health Organization (WHO), annually, nearly 11 million people worldwide need medical attention because of a burn injury and burns cause about 180,000 deaths [12]. Non-fatal burns are considered a leading cause of morbidity [12]. Many burn patients experience functional limitations shortly after burns [13]. Up to 24 months post-burn, most limitations improve, however, some remain highly prevalent in a subset of patients in the long-term, like psychological functioning [13]. Moreover, there are apparent latent limitations, like participation restrictions due to mental well-being, which tend to develop after physical symptoms subside [13–15]. In the recent Global Burden of Disease (GBD) study the burden associated with burn injuries has been included [16]. YLDs for 2017 presented by the GBD for burns were 78, 137 and 165 per 100,000 for the Netherlands, Australia and New Zealand respectively [16]. However, burn injuries are distinguished into six heterogeneous groupings, mainly based on burn size, body region involved and whether or not patients received treatment. These categories are hard to apply as a lot of detailed information is needed to form these groups [1].

Since the burden of disease is an important input for health decision-making, planning processes, and priority setting in health care [17, 18], there is an urgent need to improve the understanding of the burden due to burn injury. Thus, the first aim of our study was to adapt the refined INTEGRIS method of Haagsma et al. [10] to burn injuries (INTEGRIS-burns), including 1) generating homogenous groups of burn patients with similar health consequences, 2) deriving disability weights for these homogenous burn injury groups, and 3) empirically assessing the proportion of burn patients with a permanent disability. Our second aim was to apply this adapted INTEGRIS-burns method to calculate the non-fatal burden of disease of burns for Australia, New Zealand and the Netherlands.

## Methods

This study was conducted in two parts. Firstly, data were pooled and categorized to establish contemporary disability weights for burn injury (steps 1–3 below). Secondly, these disability weights were applied to estimate and compare the burden of disease of burn injury in three different countries (step 4).

### Data sources

Two different datasets were combined to form the dataset for the present study. The first dataset consisted of health-related quality of life (HRQL) data from 10 different European studies on HRQL in burn patients [19]. This dataset was created for an earlier study in which authors of European studies on HRQL studies were asked to provide their data to study the recovery of HRQL of burn patients [19]. The authors provided consent to use this dataset for the present study. This dataset includes a wide variety of burn patients (*n* = 1649) and time points on which HRQL was assessed, but relatively few outcomes were measured shortly (≤1 month) after burn (Table 1). Further, the European data probably include proportionally more patients with complaints as it was presumed that burn patients experiencing complaints were more willing to participate in the studies (participation bias). To improve the generalizability of our results, we included a second dataset from Western Australia. These data were included from systematically recorded outcomes from all inpatients (*n* = 1752) admitted to the burn center which provided a similar model of care in terms of access to critical care and acute surgical interventions. HRQL outcomes of patients were assessed at all planned follow-up visits including four to six weeks; three months; six months; 12 months; and, 24 months scheduled from the date of burn injury. However, when follow-up was no longer of benefit, patients were discharged and outcomes were no longer assessed, or patients self-select and do not return to ambulatory or video conference (telehealth) follow up. As a consequence, most outcomes were available up to 12 months post-burn and longer- term outcomes were only available from patients that return for follow-up visits or provide their survey responses in lieu of attending in person. Thus, patients with more extensive burns tended to provide their long-term outcomes, likely resulting in a greater proportion of patients with complaints or negative sequelae in the Western Australia data beyond one year after burn.

**Table 1:** Overview of data sets

Name/First author, year (reference)	Country	Inclusion criteria	Study population	Etiology	%TBSA burned, mean (SD)	LOS, mean (SD)	No of surgery, mean (SD)	HRQL instrument	Assessment time point(s)
Burns Service of Western Australia	Australia (Western Australia)	All inpatients admitted between 2004 and April 2019	*n* = 1756, (M: 69.7%). Mean age: 40.2 yr	Flame: 38.7% Scald: 28.4%	5.9% (9.1)	9.1 (12.4)	1.1 (1.2)	SF-36	4–6 weeks, 3, 6, 12, 24 months
Bloemen et al., 2012 [20]	The Netherlands	Surgery and TBSA full thickness burns < 15%, study wound surface area min. 10 cm^2^ and max. 300 cm^2^ (October 2007 – February 2010)	*n* = 77, (M: 57.1%). Mean age: 47.4 yr	Flame: 72.3% Scald: 15.4%	8.3% (7.7)	19.9 (15.2)	1.5 (0.9)	EQ-5D-3 L	3, 12 months
Hop et al., 2013 [21]	The Netherlands	Outpatient or admitted to a burn centre within 5 days post burn, with burns of indeterminate depth and a ≤ 20% TBSA burned (August 2011 – July 2013)	*n* = 124, (M: 69.4%). Mean age: 42.3 yr	Flame: 54.0% Scald: 24.2%	8.0% (11.9)	18.4 (24.8)	1.0 (1.5)	EQ-5D-3 L	3, 12, 24 months
Moi et al., 2006 [22] & Moi et al., 2016 [23]	Norway	All patients hospitalized for burn injury (1995–2000)	*n* = 90, (M: 83.3%). Mean age: 43.0 yr	Flame: 57.8% Scald: 24.4%	17.7% (12.8)	22.7 (20.3)	1.7 (1.9)	SF-36	Measurement 1: 11–82 months Measurement 2: 150–220 months
Orwellius et al., 2013 [24]	Sweden	Burn patients with ≥10% TBSA burned or LOS of ≥7 days (March 2000 – December 2009)	*n* = 118, (M: 77.1%). Mean age: 48.2 yr	NA	23.3% (17.6)	29.8 (32.4)	NA	EQ-5D-3 L	12 and 24 months
Oster et al., 2011 [14]	Sweden	Burn patients with ≥5% TBSA burned or LOS of > 1 day (March 2000 – March 2007)	*n* = 67, (M: 77.6%). Mean age: 42.6 yr	Flame: 74.6% Scald: 10.4%	25.6% (20.2)	26.9 (33.5)	NA	EQ-5D-3 L	Admission, 3, 6, 12, 24 months, 2–7 years (mean 4.6 yr)
Renneberg et al., 2014 [25]	Germany	All patients hospitalized in the burn unit (June 2004 and November 2006)	*n* = 292, (M: 72.3%). Mean age: 39.6 yr	NA	15.0% (14.2)	28.1 (31.2)	2.6 (4.8)	SF-36	6, 12, 24, 36 months
Spronk et al., 2019 [15]	The Netherlands	Burn patients with LOS of ≥1 day or with surgery (2010–2013)	*n* = 256, (M: 62.1%). Mean age: 47.7 yr	Flame: 57.9% Scald: 18.5%	9.6% (16.9)	17.5 (22.0)	1.3 (1.9)	EQ-5D-5 L	5–7 years (mean 5.5 yr)
Van Loey et al., 2012 [26]	Belgium and The Netherlands	Burn patients with LOS of ≥72 h (March 2003 and April 2005)	*n* = 257, (M: 72.4%). Mean age: 38.9 yr	Flame: 57.3% Scald: 24.9%	12.7% (11.5)	24.2 (23.0)	1.5 (2.2)	EQ-5D-3 L	3 weeks, 3, 9, 18 months
Hoogewerf et al., 2014 [27]	Belgium and The Netherlands	Burn patients with LOS of ≥72 h (March 2006 – January 2009)	*n* = 297, (M: 79.8%). Mean age: 40.8 yr	Flame: 65.1% Scald: 22.3%	12.9% (12.1)	22.6 (20.9)	1.1 (1.7)	SF-36	3 and 18 months
Bosmans et al., 2015 [28]	The Netherlands	Burn patients with TBSA≥1% burned or LOS ≥ 48 h (April 2010 – October 2012)	*n* = 145, (M: 65.5%). Mean age: 40.6 yr	Flame: 58.3% Scald: 30.9%	9.0% (8.0)	17.2 (13.2)	0.9 (1.5)	EQ-5D-3 L	2 weeks, 3, 6, 12, 18 months

The combined dataset included adult burn patients (≥18 years old) who were admitted to a burn center. Data was collected between 1995 and 2019, originated from Australia, Belgium, Germany, Norway, Sweden and The Netherlands, and was anonymously shared (Table 1) [19]. In all data sets, patients with cognitive impairment were excluded. In all European datasets, patients with poor language proficiency were excluded as well. All datasets were collected in accordance with the Declaration of Helsinki and written informed consent was obtained from all individual participants included in the study. This study was approved by the South Metropolitan Health Service Ethics Committee (registration number RGS2233-SP1). This data is accessed and analysed with a waiver of consent based on the proviso of presentation of summarised or aggregated data.

### Health-related quality of life measures

It has been recommended that case-based disability weights (i.e. based on self-reported patient data) should be used to more accurately quantify the burden of injuries [11]. Patient-reported outcome measures, such as the EuroQol - 5 Dimensions (EQ-5D), can be used to obtain such case-based disability weights [9]. In the data sets included, the EQ-5D (both the 3 L and 5 L versions) as well as the Medical Outcome Study Short Form - 36 items (SF-36) were used to assess patient-reported outcomes (Table 1). The SF-36 data was transformed into EQ-5D-3 L data by application of the algorithm developed by Gray et al. [29], and EQ-5D-5 L data was mapped into EQ-5D-3 L data using the method of Van Hout et al. [30]. After these transformations, data were merged into one large combined EQ-5D-3 L dataset that was used for the analyses of step 1–3.

The EQ-5D-3 L consists of five dimensions (mobility, self-care, usual activities, pain/discomfort and anxiety/depression) and a visual analogue scale (VAS) for general health. The five dimensions are scored on three levels of severity (no problems, some problems and severe problems) to describe a patient’s health state [31, 32]. These health states were converted into utility weights by use of the value set of the general population of the United Kingdom (UK) [33]. Utility weights can range between 0 (death) and 1 (full health). It can also have a negative value (minimum − 0.59) for health states worse than death. The UK value set was used as not all included countries had an own country-specific set [34]. For the calculation of the empirically derived EQ-5D-3 L disability weights, the age and sex adjusted health index of the general population of the United Kingdom was used [35].

### Step 1: homogenous severity categorization of burn patients

To determine groups of burn patients that are homogeneous in terms of health consequences, it was necessary to link incidence data and disability information as the consequences of burns can vary widely according to severity of the injury [36, 37]. Literature was studied, short- and long-term EQ-5D data from the combined dataset was assessed and experts (both clinicians and patients) were consulted to derive homogenous groups of burn patients with similar functional outcomes. Preconditions were that 1) these groups are easily identified within the total group of burn patients and 2) are based on data that are registered worldwide, so that the grouping can be widely applied among burn researchers. Based on the above described steps and with the assumption that patients are treated in a similar way and comparable resources, the next homogenous groups have been chosen that were comparable on EQ-5D-3 L outcomes: < 5% TBSA burned (or %TBSA missing), 5–20% TBSA burned and > 20% TBSA burned. The most severe group (i.e. > 20%TBSA) is in line with the criteria of the American Burn Association [38].

### Step 2: calculation of disability weights

The disability weight is the difference between the EQ-5D-3 L utility score and the corresponding sex- and age-specific norm score [10, 39]. Disability weights for the homogenous groups based on %TBSA were created by aggregating the disability weights of the individual patients. Disability weights were calculated separately for five different time periods in the recovery of burns, including four time periods in the short-term (0–1 months, > 1–6 months, > 6–12 months, > 12–24 months) and one in the long-term (> 24 months) [10]. We also calculated them separately for the European and Western Australian data in order to see whether these disability weights differed.

### Step 3: lifelong disability

The proportions of patients with lifelong disability (i.e. long-term; > 24 months) were determined for each homogenous group based on EQ-5D-3 L data exploration in the combined dataset and was validated by expert opinion (both clinicians and patients). Lifelong disability was assumed when a patient reported a severe problem (level 3 EQ-5D-3 L) in any of the five EQ-5D-3 L dimensions or mild problems (level 2 EQ-5D-3 L) for both the dimensions pain/discomfort and anxiety/depression at two-year follow-up. These two dimensions were chosen based on literature; these dimensions are most often affected by burns [13, 15]. The percentage of patients with lifelong disabilities was assessed from the combined dataset. Seventeen Dutch patients and seventeen Australian and Dutch clinicians were asked to provide their opinions about the percentage of patients with lifelong disability via a short survey in order to externally validate the dataset results. Thirteen patients and fourteen clinicians completed the survey. The mean percentages reported by the experts were compared with the percentages revealed by exploring our dataset (Appendix 1). The percentages revealed by exploring our dataset were used in the present study. These were: 20% for the patient group ≤5% TBSA burned, 25% for the group 5–20% TBSA burned and 39% for the group > 20% TBSA burned.

### Step 4: calculation of non-fatal burden of disease of burns

**Fig. 1:**
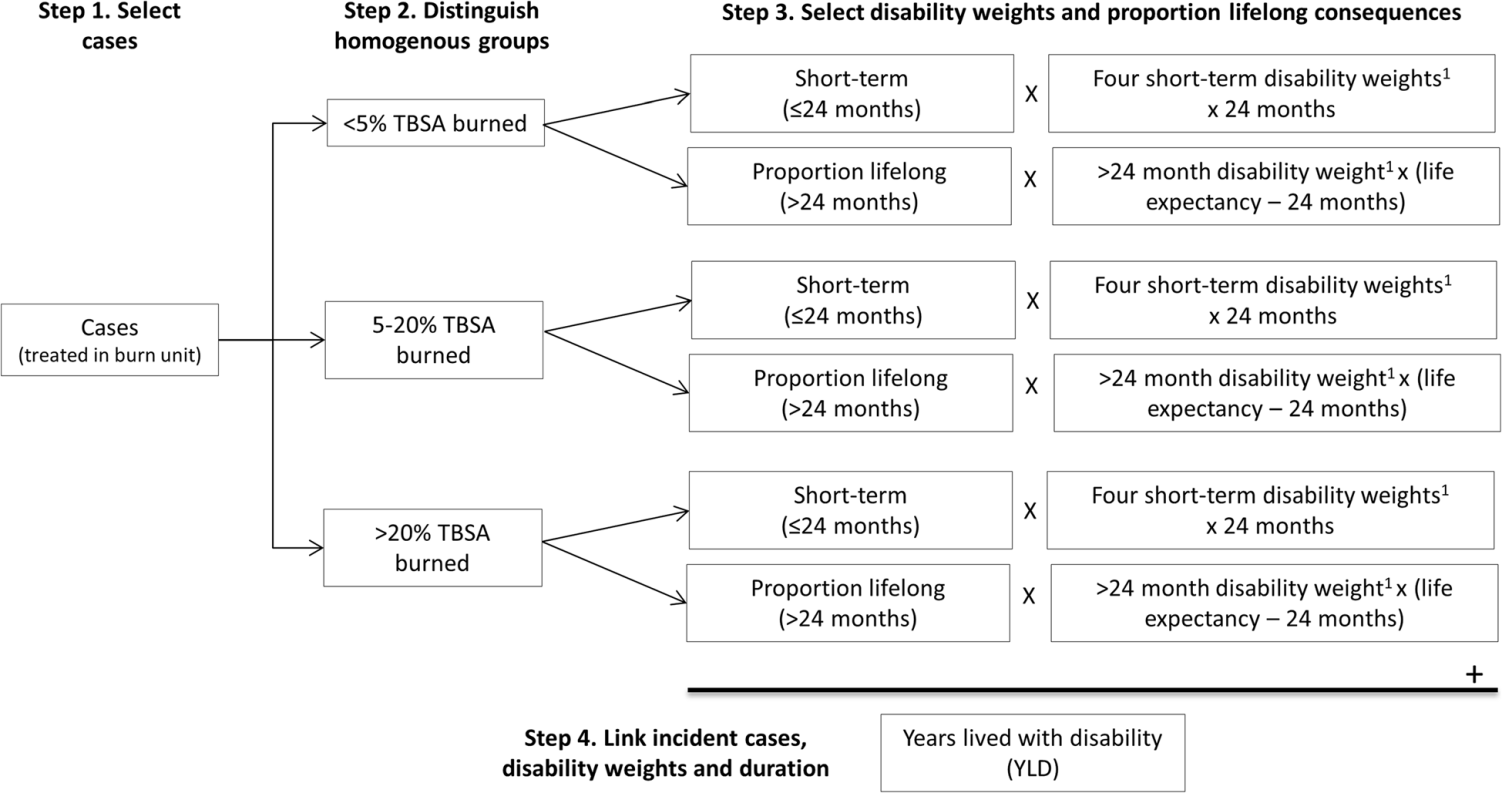
Methodology to derive years lived with disability adapted from INTEGRIS method from Haagsma et al. [10]. ^1^Disabilty weights and life expectancies are adjusted for age and sex [8]. TBSA = total body area burned

In order to calculate the non-fatal burden of disease of burns, incidence data is required. By combining the disability weights with incidence data, the non-fatal burden of disease expressed as years lived with disability (YLD) was calculated separately for the short-term (0–1 months, > 1–6 months, > 6–12 months, > 12–24 months) and the long-term (> 24 months), see Fig. 1 and Appendix 2. YLDs were calculated by applying the following formula:

$$ YLD= number\ of\ incident\ cases\ast disability\ weight\ast average\ duration\ \left( in\ years\right) $$

We have contacted several international experts to find country-level burn incidence data of burn injuries from multiple countries, including low and middle-income countries. The only incidence data that was accessible for use was the data from Australia, New Zealand and the Netherlands and therefore we used the data from these three countries to apply the refined method. Incidence data on burns from Australia, New Zealand and the Netherlands for the years 2010 to 2017 were derived from the Burns Registry of Australia and New Zealand (BRANZ) [40] and from the Dutch Burn Repository (DBR) R3 [41]. Data from BRANZ included patients that were admitted for at least 24 h to a burn centre or had surgery; data from the Dutch Burn Repository R3 included patients admitted for at least two hours to a specialized burn centre. Due to privacy reasons and potential identification of patients, BRANZ policy precludes the provision of exact incidence counts for sub-grouped cells between one and five. Therefore, we applied an assumed incidence rate of 2 as an average for these categories where specific detail was lacking. The non-fatal burden of disease was calculated for each of these three countries separately.

The calculation of YLDs for the short-term (acute phase) consisted of three steps: (i) gathering data on the incidence and age and sex distribution of burns, (ii) breaking down the incidence data into the homogenous burn categories, and (iii) combining the grouped incidence data with the relevant disability weights and durations (i.e. 0–1 month, > 1–6 months, > 6–12 months, > 12–24 months) (Fig. 1) [10]. Short-term YLDs were calculated by multiplying the disability weights by the corresponding duration over which the disability weight applied and incidence. For example, the 0–1 month disability weight was multiplied by 1/12 and by the incidence of the corresponding group; the > 1–6 month disability weight by 5/12 and by the incidence of the corresponding group.

For the long-term (i.e. after 24 months) YLD calculations it was assumed that part of the burn population experiences lifelong consequences, and that the proportions of patients that experience these lifelong consequences vary for the different homogenous burn categories. To calculate long term YLDs the grouped incidence data by category were combined with the relevant disability weights and the remaining life expectancy minus 2 years [10]. Minus two years corresponds to the time period of the short-term disability. The remaining life expectancy was derived from the GBD study [42].

Short-term and long-term YLDs were summed up to derive YLDs on homogenous group level. The YLDs from the different groups were again summed to derive the overall YLDs of burns for each of the three different countries. YLD per patient as well as the impact of YLDs on a country population level (i.e. YLDs divided by number of people registered in each of the three countries) were compared among the three different countries. Country population data was derived from the Australian Bureau of Statistics, Stats New Zealand and the Central Bureau of Statistics Netherlands [43–45].

### Data analyses

Demographics and disability weights from the combined dataset were presented and compared among the two different datasets. Mann Whitney U tests were used for comparison of continuous variables and chi-square tests for categorical variables. IBM SPSS Statistics 23 was used to perform analyses and calculations.

## Results

### Patients

The combined dataset included 3401 patients. Of these, 1649 originated from the European dataset and 1752 from the Western Australia dataset (Table 2). The patients in the combined dataset had a mean age of 41.1 years (SD 15.5) and 70.9% was male. Mean %TBSA burned was 9.6% (SD 12.2) and patients had a mean length of hospital stay (LOS) of 16.0 days (SD 20.7) and on average 1.3 surgeries. Most burns were caused by flames. Patients in the European dataset were statistically significantly older, had a higher mean %TBSA, a longer LOS and more surgical procedures (Table 2). The combined dataset included 7159 EQ-5D-3 L outcomes: 3708 outcomes were available from the European dataset and 3451 from the Western Australia dataset.

**Table 2:** Demographic characteristics of combined dataset for step 1–3

Variable	Total sample (*n* = 3401)	European (EU) sample (*n* = 1649)	Western Australian (WA) sample (*n* = 1752)	Difference between EU and WA sample
Gender				
Male, n(%)	2412 (70.9%)	1192 (72.3%)	1220 (69.6%)	*p* = 0.089
Age				
Mean (SD)	41.1 (15.5)	42.0 (14.6)	40.3 (16.3)	*p* < 0.001
Range	18–90 years	18–90 years	18–89 years	
%TBSA burned				
Mean (SD)	9.6 (12.2)	13.5 (13.7)	5.9 (9.1)	*p* < 0.001
Range	0–90%	0–90%	0–75%	
%TBSA burned				
0- < 5%	1587 (46.7%)	429 (26.0%)	1158 (66.1%)	
5–20%	1364 (40.1%)	882 (53.5%)	482 (27.5%)	
> 20%	450 (13.2%)	338 (20.5%)	112 (6.4%)	
Length of hospital stay				
Mean (SD)	16.0 (20.7)	23.1 (24.8)	9.1 (12.4)	*p *< 0.001
Range	0–246 days	0–246 days	0–130 days	
Nr of surgeries				
Mean (SD)	1.3 (2.1)	1.5 (2.7)	1.1 (1.2)	*p* = 0.013
Range	0–35 surgeries	0–35 surgeries	0–12 surgeries	
Nr surgery, n(%)				
0	720 (21.2%)	487 (29.5%)	233 (13.3%)	
1	1682 (49.5%)	608 (36.9%)	1074 (61.3%)	
> 1	569 (16.7%)	369 (22.4%)	200 (11.4%)	
Unknown	430 (12.6%)	185 (11.2%)	245 (14.0%)	
Etiology (%)				*p* < 0.001
Scald	784 (23.1%)	277 (16.8%)	507 (28.9%)	
Contact	282 (8.3%)	52 (3.2%)	230 (13.1%)	
Flame	1444 (42.5%)	753 (45.7%)	691 (39.4%)	
Chemical	155 (4.6%)	59 (3.6%)	96 (5.5%)	
Electrical	82 (2.4%)	56 (3.4%)	26 (1.5%)	
Other	129 (3.8%)	36 (2.2%)	94 (5.4%)	
Unknown	525 (15.4%)	416 (25.2%)	109 (6.2%)	

### Disability weights

The calculated disability weights are presented in Table 3. Within the subgroup < 5% TBSA burned, disability weights ranged from 0.173 (0–1 month post-burn) to 0.046 (long-term; > 24 months post-burn) in the total sample. For the subgroups 5–20% TBSA burned and > 20% TBSA burned, these ranges were 0.099–0.264 and 0.122–0.497, respectively. In general, the highest disability weights represent the recovery phase most acutely after burns, with each subsequent recovery phase having a diminishing disability weight. The only exception was the disability weight for the subgroup < 5%TBSA burned for the recovery phase > 12–24 months; this disability weight was slightly higher than the disability weights for the earlier recovery phases (Table 3).

**Table 3:** Mean disability weights for the three different homogenous groups of burn patients, by recovery period

Homogenous groups and different recovery periods	Total sample Mean disability weight (95% CI)	European sample Mean disability weight (95% CI)	Western Australian sample Mean disability weight (95% CI)	Difference between EU and WA sample
< 5% TBSA burned				
0–1 months	0.173 (0.152 to 0.195)	0.298 (0.235 to 0.362)	0.141 (0.120 to 0.161)	**< 0.001**
> 1–6 months	0.098 (0.090 to 0.106)	0.118 (0.098 to 0.139)	0.094 (0.085 to 0.103)	**0.032**
> 6–12 months	0.082 (0.067 to 0.097)	0.110 (0.080 to 0.141)	0.067 (0.051 to 0.084)	**0.007**
> 12–24 months	0.102 (0.080 to 0.123)	0.099 (0.073 to 0.126)	0.106 (0.067 to 0.144)	0.791
> 24 months	0.046 (0.026 to 0.067)	0.046 (0.026 to 0.067)	NA	
5–20% TBSA burned				
0–1 months	0.264 (0.238 to 0.289)	0.325 (0.286 to 0.363)	0.193 (0.163 to 0.224)	**< 0.001**
> 1–6 months	0.139 (0.128 to 0.150)	0.160 (0.144 to 0.176)	0.113 (0.098 to 0.129)	**< 0.001**
> 6–12 months	0.118 (0.104 to 0.133)	0.134 (0.116 to 0.152)	0.085 (0.065 to 0.106)	**0.001**
> 12–24 months	0.108 (0.092 to 0.124)	0.106 (0.089 to 0.123)	0.119 (0.071 to 0.167)	0.593
> 24 months	0.099 (0.077 to 0.122)	0.099 (0.077 to 0.122)	NA	
> 20% TBSA burned				
0–1 months	0.497 (0.438 to 0.557)	0.579 (0.515 to 0.643)	0.224 (0.142 to 0.307)	**< 0.001**
> 1–6 months	0.262 (0.235 to 0.290)	0.291 (0.254 to 0.328)	0.214 (0.172 to 0.256)	**0.009**
> 6–12 months	0.231 (0.198 to 0.263)	0.250 (0.211 to 0.288)	0.172 (0.110 to 0.233)	**0.043**
> 12–24 months	0.163 (0.134 to 0.191)	0.154 (0.126 to 0.182)	0.221 (0.102 to 0.340)	0.119
> 24 months	0.122 (0.092 to 0.152)	0.122 (0.092 to 0.152)	NA	

1056; 3238; 1355; 1001; and 509 outcomes were used to calculate the disability weights for 0–1 month, > 1–6 months, > 6–12 months, > 12–24 months, and > 24 months, respectively. Values printed in bold are statistically significant

The disability weights based on the European data and Western Australia data separately are also presented in Table 3. Disability weights up to 12 months post-burn were significantly higher when based on the European data compared to the Western Australia data. In the following recovery phase (> 12–24 months), disability weights based on the Western Australia data tended to be higher, though the difference is not significant.

### Non-fatal burden of disease of burns

The adapted INTEGRIS-burns methodology was applied to estimate the non-fatal burden of disease of burns for Australia, New Zealand and the Netherlands. Table 4 presents the estimated YLDs for both the subgroups and the total burn population for these countries for 2017. The mean burden of burns per case in Australia, New Zealand and the Netherlands in 2017 were 1.00, 1.20 and 1.04, respectively. For the different subgroups, the YLD per case ranged from 0.65 for the < 5%TBSA burned group for Australia and the Netherlands, to 2.59 for the > 20% TBSA group for Australia.

**Table 4:** Estimates of the non-fatal burden of disease expressed as years lived with disability (YLD) for the different homogenous groups of burn patients for Australia, New Zealand and the Netherlands in 2017

2017	Australia	New Zealand	The Netherlands
< 5% TBSA			
Incidence	1849	244	445
YLD	1205.7	166.5	288.2
YLD per case	0.65	0.68	0.65
YLD total population per 100,000	122.5	91.4	48.3
5–20% TBSA			
Incidence	839	143	259
YLD	1217.0	233.8	402.0
YLD per case	1.45	1.63	1.55
YLD total population per 100,000	119.6	132.6	75.7
> 20% TBSA			
Incidence	162	48	40
YLD	419.2	122.0	80.4
YLD per case	2.59	2.54	2.01
YLD total population per 100,000	38.8	55.2	9.3
Total			
Incidence	2850	435	744
YLD	2841.9	522.2	770.6
YLD per case	1.00	1.20	1.04
YLD total population per 100,000	280.9	279.2	133.3

**Fig. 2:**
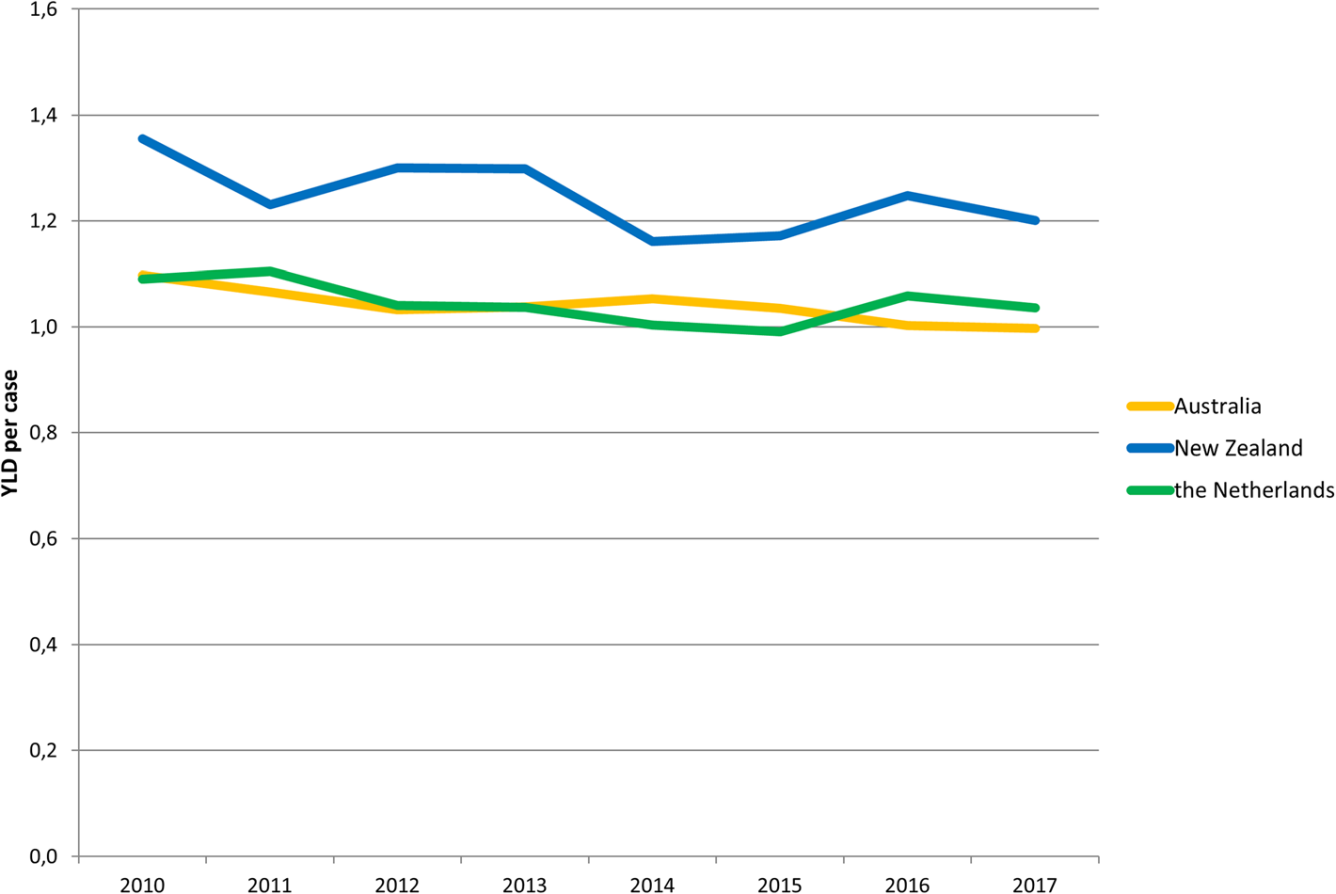
Estimates of the non-fatal burden of disease expressed as years lived with disability (YLD) per case for Australia, New Zealand and the Netherlands in 2010 to 2017

The YLDs per case over time in the three countries seem to slightly decrease over time between 2010 and 2017 (Fig. 2). Highest values in the YLDs per case were seen for New Zealand. YLDs per case ranged between 1.10 in 2010 and 1.00 in 2017 for Australia; between 1.36 in 2010 and 1.20 in 2017 for New Zealand; and between 1.10 in 2010 and 1.04 in 2017 for the Netherlands.

**Fig. 3:**
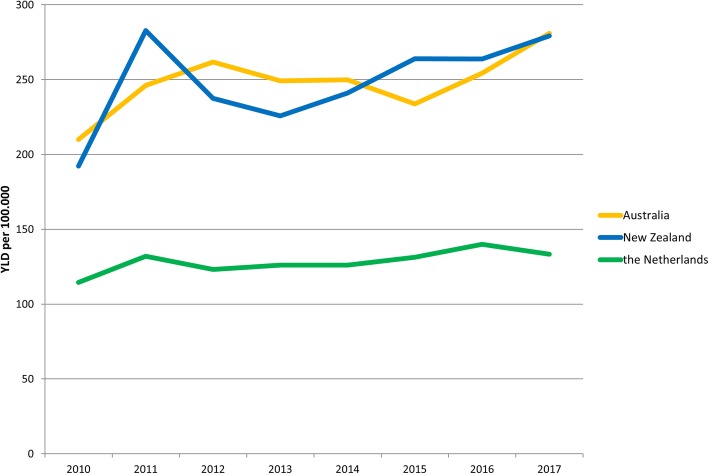
Estimates of the non-fatal burden of disease expressed as years lived with disability (YLD) for the total population of Australia, New Zealand and the Netherlands in 2010 to 2017**.** *Note.* Not all burn units in Australia were contributing until 2017; and not all burn units in New Zealand were contributing until 2013 [46]

**Fig. 4:**
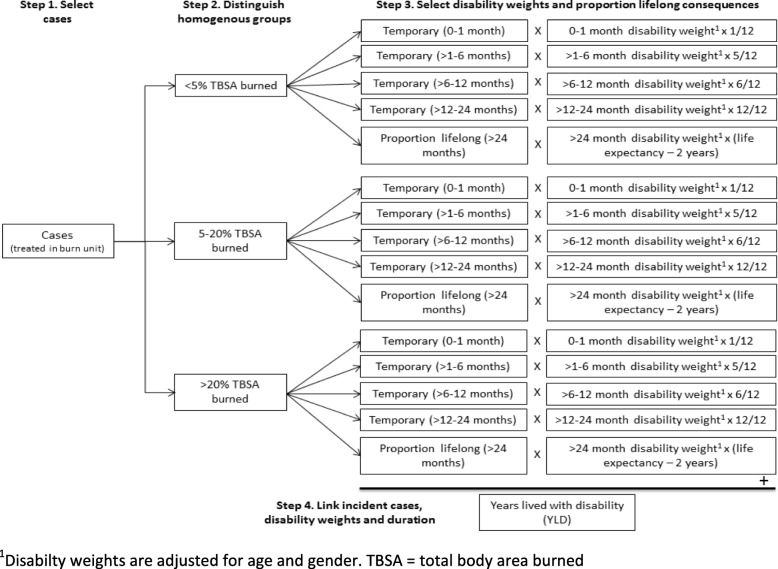
Methodology to derive years lived with disability adapted from the INTEGRIS method developed by Haagsma et al. [10]. ^1^Disabilty weights are adjusted for age and gender. TBSA = total body area burned

The burden of disease of burns for the three countries in total for 2017 is also presented in Table 4. The YLDs for the total population per 100,000 persons was 280.9 for Australia, 279.2 for New Zealand and 133.3 for the Netherlands. Over time, YLDs for the total population seem to slightly increase in the three countries (Fig. 3). The YLDs for the total population ranged from 210.1 in 2010 to 280.9 in 2017 for Australia, from 192.2 in 2010 to 279.2 in 2017 for New Zealand, and from 114.5 in 2010 to 133.3 in 2017 for the Netherlands.

## Discussion

The burden of disease is an important resource in public health and there was an urgent need to adapt a refined method to estimate of the burden of disease for burn injuries. Three homogenous groups with respect to health consequences have been created based on %TBSA burned. A set of 12 short-term disability weights (four for each homogenous group) and three life-long disability weights were derived and presented. The time point after which we consider disabilities caused by burns as either resolved or permanent was assumed to be 24 months, instead of 12 months which was used in all earlier methods. We would argue that post-burn sequelae, including scar symptoms and mental health challenges, are frequently measurable in the period beyond one year after injury [13, 47]. The proportion of burn patients with lifelong disabilities was defined based on exploration of HRQL data and externally validated by a group of experts. The proportions of patients with lifelong disabilities included 20% for the patient group ≤5% TBSA burned, 25% for the group 5–20% TBSA burned, and 39% for the group > 20% TBSA burned. Application of the INTEGRIS-burns method showed that the YLD per case decreased over time from 2010 to 2017 in Australia, New Zealand and the Netherlands, whereas the YLD per 100,000 inhabitants tends to increase, particularly due to the increasing incidence of burns in all three countries.

Our method created groups of burn patients with homogenous outcomes that are easy identified within burn data. Hereby, this method is easy to apply (i.e. only sex, age and %TBSA burned of a burn population is needed) and usable in settings with limited resources. Besides, the definition of the most severe group (i.e. > 20%TBSA) is in line with the criteria of the American Burn Association [38]. We have considered other variables and more specific groups, but that makes the method more difficult to apply. Another disadvantage of more specific groups is the influence of a health care system or treatment strategies on the characteristics applied. For example, there is evidence that HRQL and disability after burns are related with length of hospital stay [13, 36, 37]. However, length of hospital stay can be influenced by the treatment strategy (i.e. an early excision strategy vs a conservative approach), the parameters of the catchment area, therapeutic supports outside the burn centre and other non-treatment related issues like policies (i.e. coverage of health care costs) or logistic issues [48, 49], which make this characteristic not an universal applicable variable. Other methods also used body region affected and lower airway burns to group burn patients [1, 39]. No convincing evidence exists on either of these variables and therefore these variables were not considered [36].

Two other important differences between our method and the existing methods is the breakdown of recovery after burns and the use of 24 months as the time point on which disabilities caused by burns are either resolved or permanent. According to the recovery of HRQL in burn patients [13, 19], we derived four short-term disability weights up to 24 months for each homogenous group, whereas other methods only provide one 12-month disability weight per group. Earlier methods used 12 months as time point to consider a disability lifelong, even though, it is shown that 12-months is too short for the recovery of burns and for the maturation of scars [13, 50]. That 12 months should be reconsidered for some other injuries was also highlighted by Gabbe et al., they stated that 12 months would not be suitable for all injuries as some have a longer recovery phase or late improvements [39, 51].

The application of different subgroups, a different time point on which disabilities were considered lifelong, and disability weights derived for more detailed periods in the recovery of burns, hamper comparison of our disability weights with those from earlier studies [1, 10, 39]. However, when roughly comparing the disability weights, the short-term disability weights from the present study were in about the same range as the short-term disability weights from other studies for burns [1, 10, 39], except for the disability weights 0–1 month after burns. Long-term disability weight from earlier studies were defined as > 12-month disability weights and ranged between 0.019 and 0.110 for burns < 20% TBSA, which were comparable to the > 24-month disability weights from present study for the subgroups < 5%TBSA and 5–20% TBSA. Long-term > 12-month disability weights presented by earlier studies were between 0.156 and 0.161 for burns ≥20% TBSA [1, 39], whereas the long-term 24-month disability weight from our study for the group > 20%TBSA was somewhat lower, most probably due to the cut-off point of 24 months instead of 12 months.

In the present study, the highest disability weights represent the recovery period most acutely after burns, with each following time period having a reduced disability weight. The only exception was the disability weight for the subgroup < 5%TBSA burned for the recovery period > 12–24 months; this disability weight was slightly higher than the disability weights for the earlier recovery periods. This exception can be induced by selective (lost to) follow-up. The retention rate may have been higher among patients that continue having complaints in the European studies; and patients who perceive that there is benefit in attending assessments and treatment for their long-term sequelae, remain in the Western Australia follow-up burn care service beyond one year. On the other hand, the slightly higher disability weight for the recovery period > 12–24 months in the < 5% TBSA group may also be a true consequence of burns. Earlier studies showed that some domains of HRQL worsen in the longer-term, as well as body image and social participation [13, 52].

Through the use of 24 months as cut-off point for lifelong disability, we also had to investigate the proportion of patients that were considered having lifelong disabilities in each group. Proportions were determined based on data exploration and these were validated by both clinicians and patients. The only other method that presents proportions of patients with lifelong consequences is the GBD study [1]. This study presents a range of probability of long-term disability outcome [53] for two subgroups at 12 months, with an average of 50% of the patients < 20% TBSA and 22% of the patients ≥20%TBSA with long-term disability outcomes. This seems to be contradictory to all literature and our results that more severe burns (higher %TBSA) are associated with a higher risk of long-term consequences [36].

We applied the adapted method to estimate the non-fatal burden of disease of burns in Australia, New Zealand and the Netherlands. Non-fatal burden of disease estimates from our study are much higher compared to the non-fatal burden of disease estimates from the GBD study. YLDs for 2017 presented by the GBD for burns were 78, 137 and 165 per 100,000 for the Netherlands, Australia and New Zealand respectively [16]. The YLDs estimates based on our adapted method lie 1.7 to 2.1 times higher. This is in line with the results presented by Haagsma et al. for injuries in general; compared to the conventional methods, estimates from the refined method were 3 to 8 times higher [10]. The high differences in non-fatal burden of disease estimates for the three countries are particularly induced by on the one hand the incidence rates, with the highest incidence rates for Australia. On the other hand, the proportion of patients with major burns (> 20%TBSA burned) in each of the populations plays a role; New Zealand had the highest proportion of patients with major burns per capita which is reflected in the YLDs per case and subsequently the total YLD estimate.

### Strengths and limitations

This study has a number of strengths and limitations. Strengths include the large combined dataset and the detailed approach used to adapt the refined methodology. We combined both study related and systematically recorded outcome data to compose a large and representative dataset, including outcomes from six countries and over 3000 burns patients, to derive the disability weights. This gave us the opportunity to study groups with arguably more homogenous outcomes, to derive disability weights for five different time periods during the recovery of burns for each group, and to study the proportion of patients in each group that has permanent disability. A limitation is the transformation of HRQL data to derive one dataset. We applied the algorithm of Gray et al. [29] to transform SF-36 data to EQ-5D data which has shown to have moderate to good ability to estimate EQ-5D scores [54] and this method was used before for the purpose of deriving disability weights [39]. Another limitation is that incidence rates of the different registries included different case definitions; data from BRANZ included patients that were admitted for at least 24 h to a burn centre or had surgery; data from the Dutch Burn Repository R3 included patients admitted for at least two hours to a specialized burn centre. And, in contrast to Australia and New Zealand, in the Netherlands not all burn patients with small burns are admitted to a burn centre and only those admitted to a burn centre are included in the incidence rate, which might have led to an underestimation of the YLDs in the Netherlands. Also, due to privacy requirements, BRANZ did not provide incidence counts for sub-grouped cells between one and five patients. Therefore, we used an incidence rate of 2 as an average for these categories. This could have influenced the YLDs presented for Australia, and particularly New Zealand as many low numbers were presented for the New Zealand population. Besides, not all burn units in Australia were contributing until 2017; and not all burn units in New Zealand were contributing until 2013 which has influenced the trends presented [46]. Another limitation is the inclusion of only immediate health consequences in the YLD calculation. Recent studies by Duke et al. showed that burn patients have an increased risk of developing cardiovascular, gastrointestinal, nervous system and infectious diseases [55–59]. These more delayed consequences of burns should be ideally captured in our incidence-based burden of disease approach, however, no data on such consequences was available. Future studies should incorporate both immediate and delayed health consequences to estimate the burden of disease due to burns more completely. Another limitation is that we solely included data from high-income countries. We have contacted several international experts to find data from low and middle-income countries, but we did not manage to access data that could be used in the present study. The use of data from only high-income services makes it unclear whether this method can be applied in low and middle-income countries. It is highly likely that disability weights will be significantly different, when considering the impact of lack of access to health resources and reliable acute services, such as surgery and mechanical ventilation. Earlier studies have shown that there are differences among countries and cultures on how people perceive health problems and on how they find that these problems affect their daily activities [60, 61]. Future studies should focus on whether the application of disability weights from high-income countries is valid in low- and middle- income countries, as the vast majority of burn injuries occur in low- and middle-income countries [62].

## Conclusion

This project established a method for more precise estimates of the YLDs of burns, as it is the only method adapted to the nature of burn injuries and their recovery. Compared to previous used methods, the INTEGRIS-burns method includes improved disability weights based on severity categorization of burn patients; a better substantiated proportion of patients with lifelong disability based; and, the application of burn specific recovery timeframes. Information derived from the adapted method can be used as input for health decision making at both the national and international level. Differences between YLDs of the countries studied show that it is important to use national (or perhaps regional) data to estimate the burden of disease of burns.

## Data Availability

The European datasets used and/or analysed during the current study are available from the specific author (see Table 1) on reasonable request. For Australian data, ethical approval is needed for each specific study. Data can be requested from Dr. Dale Edgar (Dale.Edgar@health.wa.gov.au) and ethic approval from the South Metropolitan Health Service Ethics Committee (smhs.hrec@health.wa.gov.au; www.southmetropolitan.health.wa.gov.au). Secondary data from Australia and New Zealand is publically available from https://www.monash.edu/medicine/sphpm/branz/publications-and-reports. Secondary data from the Netherlands can be accessed with permission from baarm@maasstadziekenhuis.nl.

## Appendix 1

**Table 5:** Proportions of patients with lifelong consequences

	< 5% TBSA	5–20% TBSA	> 20% TBSA
	Mean	Mean	Mean
Data driven approach: Proportion of sample with a severe problem in any of the five EQ-5D dimensions or mild problems at both the dimensions pain/discomfort and anxiety/depression at 24 months	20%	25%	39%
Expert opinion: Patients (*n* = 13)	14%	24%	52%
Expert opinion: Clinicians (*n* = 14)	19%	31%	58%

## Abbreviations

%TBSA: Percentage total body surface area
BRANZ: Burns Registry of Australia and New Zealand
DALY: Disability adjusted life year
DBR: Dutch Burn Repository
EQ-5D: EuroQol - 5 Dimensions
HRQL: Health-related quality of life
SF-36: Medical Outcome Study Short Form - 36 items
UK: United Kingdom
VAS: Visual analogue scale
YLD: Years lived with disability
